# Long-term efficacy of anti-IL-4 receptor antibody in a patient with aspirin-exacerbated respiratory disease and IgG4-related disease

**DOI:** 10.1186/s13223-023-00825-z

**Published:** 2023-08-05

**Authors:** Hyun-Seob Jeon, Jae-Hyuk Jang, Youngsoo Lee, Hae-Sim Park

**Affiliations:** Department of Allergy and Clinical Immunology, Ajou University School of Medicine, Suwon, Gyeonggi-do 16499 South Korea

**Keywords:** Dupilumab, AERD, Severe asthma, Chronic rhinosinusitis, nasal polyps, IgG4, IL-4 receptor, Type 2 airway inflammation

## Abstract

**Background:**

Aspirin-exacerbated respiratory disease (AERD) and IgG4-related disease (IgG4RD) share a common pathway of Th2-mediated immune mechanism; there have been several cases of IgG4RD developed in patients with asthma, especially in those comorbid with chronic rhinosinusitis (CRS). IgG4RD has often been treated with systemic corticosteroids, rituximab, or immune-suppressive agents, but frequently failed with relapse.

**Case presentation:**

Here, we present a case of a 64-year-old male patient with severe AERD with CRS complicated with IgG4RD, who has been successfully treated and maintained with anti-IL-4 receptor antibody, dupilumab after achieving unsatisfactory responses with previous treatments including steroids, rituximab, omalizumab, and reslizumab. The patient’s symptoms (periorbital swelling and asthmatic/nasal symptoms) were remarkably improved; serum levels of IgG4/IgE as well as plasmablast/eosinophil counts progressively decreased without any recurrence sign for over 2 years of dupilumab treatment.

**Conclusion:**

These findings demonstrate that blocking the IL-4/IL-13 pathway with dupilumab can be an effective treatment with long-term safety in patients with severe AERD with CRS complicated by IgG4RD.

## Background

Aspirin-exacerbated respiratory disease (AERD) is a distinct phenotype of asthma that is defined by Samter’s triad; the presence of asthma, chronic rhinosinusitis with nasal polyps (CRSwNPs), and hypersensitivity to cyclooxygenase 1 inhibitors [1]. It is characterized by eosinophilic inflammation found in both upper and lower respiratory tracts, which is accompanied by increased levels of cysteinyl leukotrienes, and underlying type 2 airway inflammation [2]. AERD patients suffer from chronic lower respiratory and nasal symptoms despite treatment with inhaled corticosteroids plus long-acting beta 2 agonist (ICS-LABA) and/or additional leukotriene receptor antagonists (LTRA), and even aspirin desensitization, but the emergence of biologics has shown a potent and promising therapy for effective control of upper and lower respiratory symptoms. Biologics, including anti-IgE, anti-IL-5/IL-5 receptor, and anti-IL-4 receptor antibodies, approved to be effective in this country, and other biologics, such as anti-TSLP, are not available.

IgG4-related disease (IgG4RD) is a pseudo-tumorous disease accompanied by inflammation and fibrosis with single or multiple organs involved [3]. It has been recognized since the early 2000s, presented with lymphoplasmacytic infiltrate of IgG4-positive plasma cells, followed by fibrosis with storiform pattern in tissues. The pathogenesis and pathophysiology of IgG4RD remain controversial, but the expansion of plasmablasts that produce IgG4 is thought to be driven by Th2 activity. Patients usually appear with tumorous features or swellings, especially in the lacrimal and salivary glands, and frequently show remission and reactivation. Serum IgG4 levels and plasmablast counts are thought to be useful tools to diagnose and monitor IgG4RD. Glucocorticoids (prednisolone 0.4–1 mg/kg/day) are the treatment of choice, and rituximab or immune-suppressive agents, such as azathioprine, cyclophosphamide, mycophenolate mofetil, are alternatives to spare steroid dose required. Although initial treatment often offers a rapid resolution of symptoms, the risk of recurrence is high.

Dupilumab is a human monoclonal antibody against the IL-4 receptor α-subunit that inhibits both IL-4 and IL-13 in the type 2 immunity pathway. IL-4 often contributes to the development of B cells and IgE/IgG4 production, and IL-13 induces fibrosis. Dupilumab is found to be effective in allergic diseases including atopic dermatitis, severe type 2 asthma, and CRSwNPs. The efficacy and safety of dupilumab in Korean patients with uncontrolled moderate-to-severe asthma has recently been demonstrated through the subgroup analysis of the QUEST study [4]. It is also thought to be a potential option in IgG4RD, but only a few cases of IgG4RD treated with dupilumab have been reported so far, with controversial efficacy and incomplete discontinuation of glucocorticoid therapy [5–8].

Both AERD and IgG4RD are difficult to treat and control symptoms, but it is even harder when combined together. There are several case reports showing the coexistence of asthma and IgG4RD [6–11], but to date, there has been 1 case report of IgG4RD associated with AERD [11], and there has been no published report of IgG4RD developed, and long-term efficacy/safety of dupilumab treatment in an AERD patient. Here, we represent a case of a patient with both AERD and IgG4RD, refractory to previous treatments, but had been well controlled with regular dupilumab treatment for over 2 years.

## Case presentation

A 64-year-old Korean male visited the outpatient department of Allergy and Clinical Immunology in December 2014, with the symptom of recurrent periorbital swelling whenever the dose of oral corticosteroid was reduced as well as uncontrolled asthmatic symptoms and persistent nasal obstruction. The patient was diagnosed with asthma in 2002, underwent allergen immunotherapy for 3 years, and did not use inhaled corticosteroids regularly due to voice changes and intranasal corticosteroids due to complications including nasal bleeding. Therefore, he had maintained LTRA and antihistamines, and taken oral corticosteroids frequently, whenever his symptoms were exacerbated. Both periorbital swelling, which developed around 2010 and had been managed in previous hospitals with corticosteroid treatment, was noted on physical examination. Orbit MRI suggested IgG4RD involving lacrimal glands, bilateral lateral rectus muscles, and the nasal cavity along with CRSwNPs. Biopsy of nasal tissue, including NPs, was done together with middle meatal antrostomy. Biopsy and immunohistochemistry showed up to 100 IgG4-positive plasma cells per high power field and an IgG4+ /IgG+ cell ratio of about 50%. The patient started 60 mg/day of oral prednisolone as well as anti-asthmatic medications including ICS, but symptoms were partly improved. Moreover, whenever the prednisolone was tapered below 30 mg/day, his symptoms recurred and he was brought to our hospital for further management.

**Fig. 1:**
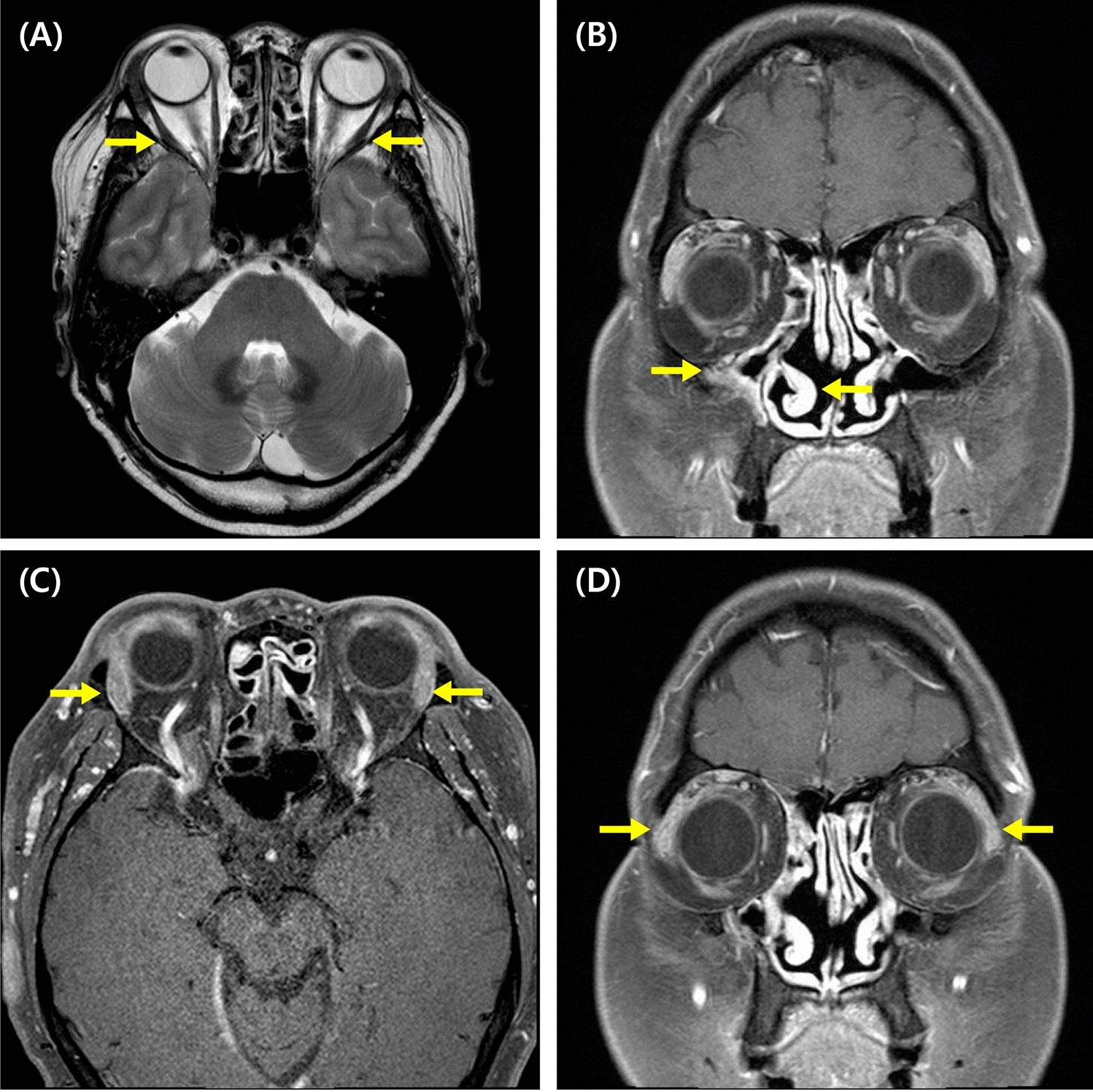
Magnetic resonance imaging of the patient before dupilumab treatment. **A** Bilateral lateral rectus muscle enhancement, **B** right maxillary sinusitis, **C**, **D** bilaterally enlarged and enhanced lacrimal glands. The corresponding lesions are marked by yellow arrows

**Fig. 2:**
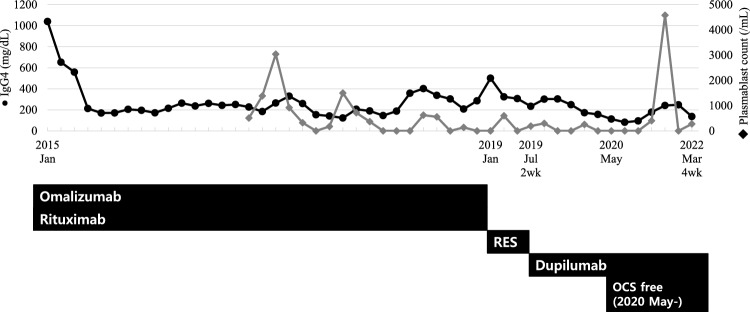
Changes in serum IgG4 levels (black circle) and peripheral plasmablast counts (grey diamond) during the treatment course. Dupilumab was initially started at 2 weeks-intervals and maintained a 4 weeks-intervals. The patient was able to become free of oral corticosteroid (OCS) from May 2020. RES: Reslizumab; OCS: oral corticosteroid

**Fig. 3:**
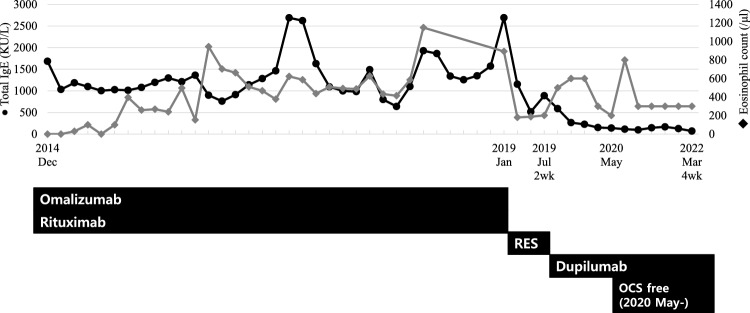
Changes in serum total IgE levels (black circle) and blood eosinophil counts (grey diamond) during the treatment course. RES: Reslizumab; OCS: oral corticosteroid

The patient was taking oral prednisolone 30 mg/day at the time of the initial visit. Periorbital swelling was present, and the patient had histories of recurrent hypersensitivity reactions to nonsteroidal anti-inflammatory drugs (NSAIDs) and a history of anaphylaxis to midazolam. The levels of serum IgG4 and IgE were 1040 mg/dL (reference range, 3.9–86.4 mg/dL) and 1684 kU/L at the initial evaluation. Orbit MRI showed consistent findings with CRS and IgG4RD, similar to the result of MRI taken in 2013 (Fig. 1). First, the dose of oral corticosteroid (methylpredinisolone) was increased to 40 mg/day. Omalizumab and rituximab were started with 150–300 mg of omalizumab at 4–8 weeks intervals, and 7 cycles of rituximab (1000 mg given twice, 2 weeks apart) were administered for 4 years until January 2019. Initially, periorbital swelling, sputum, and rhinitis symptoms seemed to subside, and the patient even had symptom-free periods without oral corticosteroids for about 2 months in January 2016. However, symptoms soon developed again, and methylprednisolone could not be reduced below 3 mg/day, making the patient dependent on oral corticosteroid. The serum level of IgG4 quickly decreased and formed a plateau level around 200 mg/dL after starting omalizumab and rituximab, but did not further decrease below that level and later started to increase again before changing to reslizumab (Fig. 2). Serum level of IgE also decreased to a plateau level of around 1000 kU/L, but showed more fluctuations and also increased later before changing to reslizumab (Fig. 3). Although blood eosinophil counts were low in the initial treatment phase, it gradually increased and showed peak level of 1200/μL before changing to reslizumab. 100 mg of reslizumab was given for 4 consecutive times as an alternative to control eosinophilia and type 2 inflammation, but the patient’s symptoms were not much improved.

Then, dupilumab was administered, starting from 300 mg every 2 weeks. Periorbital swelling and airway symptoms improved rapidly in 8 weeks after dupilumab was started. As symptoms improved, the patient could stop oral corticosteroid completely without aggravation of symptoms, and the interval of dupilumab injection was gradually extended to 8 weeks. Around this time, the serum levels of IgG4 and IgE decreased to 82.5 mg/dL and 97 KU/L, respectively, and plasmablast counts remained 0/mL and eosinophil counts estimated around 300/μL. Currently, symptoms are controlled after 4-week administration of dupilumab, and the patient remains completely free of corticosteroid after the start of dupilumab treatment (Figs. 2 and 3).

## Discussion and conclusions

In this case report, we present a case of AERD with CRSwNPs complicated with IgG4RD, showing refractory to systemic corticosteroid, omalizumab, rituximab, and reslizumab treatments, but was successfully treated and had been maintained in a well-controlled status after dupilumab treatment for over 2 years, with stopping systemic corticosteroid.

Although the pathogenesis of IgG4RD is still unknown, the prolonged, severe type 2 inflammation in the airway of an AERD patient can become a possible explanation for the development of IgG4RD. IgG4-secreting plasma cells are formed when IL-10 is introduced to B cells under the environment of type 2 inflammatory cytokines, especially IL-4 [12]. IgG4 production also requires longitudinal and repeated exposure to allergens, whether from natural exposure or from allergen-specific immunotherapy [13]. However, a recent study demonstrated that the longer disease duration in AERD patients led to higher levels of IgG4 and IL-10 expression in the CRS tissue than in those without AERD [14], suggesting close associations between AERD and IgG4RD.

The chronic type 2 inflammation in the nasal airway of AERD patients results in tissue damage, which provides immunes cells with increased exposure to allergens, microbial components, environmental factors, and also self-antigens. These stimuli can induce the formation of focal ectopic lymphoid aggregates known as tertiary lymphoid organs (TLOs), and the lack of integrity of these TLOs are thought to contribute to the development of inflammation and local autoantibody production. TLOs were also found in the nasal tissue of CRSwNP patients [15], and AERD patients showed to contain increased autoantibodies in their NP tissues [16]. This indicates that the inflammation of AERD is not only characterized as type 2 specific, but also a persistent and endless loop of inflammation triggered by various stimuli. Thus, the elevated IL-10 and IgG4 expression with the continuous loop of inflammation in nasal tissues of AERD patients may provide an ideal environment for B cells to expand and differentiate into IgG4-secreting plasma cells, resulting in the development of IgG4RD. As for the patient in this case, interrupting the IL-4 signal by dupilumab blocked both the IgG4 production pathway and the persistent inflammation of AERD, ending up to control the inflammation of IgG4RD and enabling the patient to become free of corticosteroids.

The combination of rituximab and omalizumab may have suppressed the initial immune response. However, the plasmablast count was not controlled effectively, and the levels of serum IgG4 and IgE, and the blood eosinophil count gradually increased after a long-term use of rituximab and omalizumab. This might indicate that the combination of these monoclonal antibody treatment was not sufficient as much as dupilumab was to control both the type 2 inflammation and the expansion of IgG4 producing plasmablasts. Since IL-4 is thought to be involved in the mechanisms of both AERD with CRS/NP and IgG4RD, dupilumab became the next option to control upper and lower airway inflammation in both diseases. On the other hand, dupilumab at 4 week-intervals showed effective suppression of plasmablast counts and reduced the levels of serum IgG4 and IgE, suggesting additional treatment with dupilumab as well as ICS-LABA plus LTRA can control both type 2 inflammation and IgG4 production by blocking the IL-4 pathway. In addition to the efficacy of dupilumab, safety of dupilumab without harmful adverse effects, such as a risk of infections, that can be found in rituximab is another reason that dupilumab can be a better option.

Although the patient has shown good response to dupilumab, unfortunately the injection interval of dupilumab could not be extended for more than 4 weeks. Further extended studies are needed to determine whether it is possible to extend injection intervals and even reaching remission.

In conclusion, we first report a case of IgG4RD complicated in an AERD patient with CRSwNP that was well controlled and maintained with dupilumab treatment without any long-term safety. Not only this case represented the clinical safety of long-term use of dupilumab in IgG4RD, it could also provide supplementary evidence that IL-4 and type 2 inflammation contributes to the pathogenesis of IgG4RD.

## Data Availability

Not applicable.

## Abbreviations

AERD: Aspirin-exacerbated respiratory disease
CRS: Chronic rhinosinusitis
CRSwNPs: Chronic rhinosinusitis with nasal polyps
ICS-LABA: Inhaled corticosteroids plus long-acting beta 2 agonist
IgG4RD: IgG4-related disease
LTRA: Leukotriene receptor antagonists
NSAIDs: Nonsteroidal anti-inflammatory drugs
TLOs: Tertiary lymphoid organs
TSLP: Thymic stromal lymphopoietin

## References

[1] Badrani JH, Doherty TA (2021). Cellular interactions in aspirin-exacerbated respiratory disease. Curr Opin Allergy Clin Immunol.

[2] Rhyou HI, Nam YH, Park HS (2022). Emerging biomarkers beyond leukotrienes for the management of nonsteroidal anti-inflammatory drug (NSAID)-exacerbated respiratory disease. Allergy Asthma Immunol Res.

[3] Carballo I, González-Quintela A, Sopeña B, Vidal C (2021). Immunoglobulin G4-related disease: what an allergist should know. J Investig Allergol Clin Immunol.

[4] Rhee CK, Park JW, Park HW, Cho YS (2022). Effect of dupilumab in Korean patients with uncontrolled moderate-to-severe asthma: a LIBERTY ASTHMA QUEST sub-analysis. Allergy Asthma Immunol Res.

[5] Michailidou D, Schwartz DM, Mustelin T, Hughes GC (2021). Allergic aspects of IgG4-related disease: implications for pathogenesis and therapy. Front Immunol.

[6] Nakajima I, Taniguchi Y, Tsuji H, Mizobuchi T, Fukuda K (2022). Therapeutic potential of the interleukin-4/interleukin-13 inhibitor dupilumab for treating IgG4-related disease. Rheumatology.

[7] Simpson RS, Lau SKC, Lee JK (2020). Dupilumab as a novel steroid-sparing treatment for IgG4-related disease. Ann Rheum Dis.

[8] Otani T, Iwamoto H, Yoshida Y, Yamaguchi K, Sakamoto S, Horimasu Y (2022). Dupilumab as an adjunct treatment for a patient with steroid-dependent immunoglobulin G4-related disease complicated by asthma: a case report. J Asthma.

[9] Lee YS, Cho HJ, Yoo HS, Shin YS, Park HS (2014). A case of IgG4-related disease with bronchial asthma and chronic rhinosinusitis in Korea. J Korean Med Sci.

[10] Baqir M, Garrity JA, Vassallo R, Witzig TE, Ryu JH (2016). Asthma and orbital immunoglobulin G4-related disease. Ann Allergy Asthma Immunol.

[11] Johal K, Welch K, Peters A (2017). Immunoglobulin G4 sinusitis in association with aspirin-exacerbated respiratory disease. Am J Rhinol Allergy.

[12] Lin AA, Freeman AF, Nutman TB (2018). IL-10 indirectly downregulates IL-4-induced IgE production by human B cells. ImmunoHorizons.

[13] Aalberse RC, Stapel SO, Schuurman J, Rispens T (2009). Immunoglobulin G4: an odd antibody. Clin Exp Allergy.

[14] Buchheit KM, Dwyer DF, Ordovas-Montanes J, Katz HR, Lewis E, Vukovic M (2020). IL-5Rα marks nasal polyp IgG4- and IgE-expressing cells in aspirin-exacerbated respiratory disease. J Allergy Clin Immunol.

[15] Lau A, Lester S, Moraitis S, Ou J, Psaltis AJ, McColl S (2017). Tertiary lymphoid organs in recalcitrant chronic rhinosinusitis. J Allergy Clin Immunol.

[16] Tan BK, Li QZ, Suh L, Kato A, Conley DB, Chandra RK (2011). Evidence for intranasal antinuclear autoantibodies in patients with chronic rhinosinusitis with nasal polyps. J Allergy Clin Immunol.

